# COVID-19 mortality rate and its determinants in Ethiopia: a systematic review and meta-analysis

**DOI:** 10.3389/fmed.2024.1327746

**Published:** 2024-02-27

**Authors:** Temesgen Gebeyehu Wondmeneh, Jemal Abdu Mohammed

**Affiliations:** Department of Public Health, College of Medical and Health Science, Samara University, Semera, Ethiopia

**Keywords:** COVID-19, mortality, Ethiopia, rate, risk factors

## Abstract

**Background:**

The COVID-19 mortality rate continues to be high in low-income countries like Ethiopia as the new variant’s transmission expands and the countries’ limited capacity to combat the disease causes severe outcomes, including deaths. The aim of this study is to determine the magnitude of the COVID-19 mortality rate and its determinants in Ethiopia.

**Methods:**

The main electronic databases searched were PubMed, CINAHL, Google Scholar, and African journals online. The included studies’ qualities were assessed independently using the Newcastle-Ottawa scale. The data was extracted in Microsoft Excel spreadsheet format. The pooled effect size and odds ratios with 95% confidence intervals across studies were determined using the random-effects model. I^2^ is used to estimate the percentage of overall variation across studies due to heterogeneity. Egger’s test and funnel plot were used to find the published bias. A subgroup analysis was conducted. The effect of a single study on the overall estimation was determined by sensitivity analysis.

**Results:**

A total of 21 studies with 42,307 study participants were included in the final analysis. The pooled prevalence of COVID-19 mortality was 14.44% (95% CI: 10.35–19.08%), with high significant heterogeneity (I^2^ = 98.92%, *p* < 0.001). The risk of mortality from COVID-19 disease was higher for patients with comorbidity (AHR = 1.84, 95% CI: 1.13–2.54) and cardiovascular disease (AHR = 2, 95% CI: 1.09–2.99) than their counterparts without these conditions.

**Conclusion:**

A significant number of COVID-19 patients died in Ethiopia. COVID-19 patients with comorbidities, particularly those with cardiovascular disease, should receive special attention to reduce COVID-19 mortality.

**Systematic review registration:**

https://www.crd.york.ac.uk/PROSPERO/, registration identifier (ID) CRD42020165740.

## Background

The Latin word “corona,” which means crown, is where the word “coronavirus” comes from. The name refers to the virus’ distinctive appearance under an electron microscope, which consists of rounded particles with a rim of protrusions like the solar corona (1). The severe acute respiratory syndrome coronavirus 2 (SARS-CoV-2) is the pathogen responsible for the extremely contagious disease known as coronavirus disease 2019 (COVID-19). Its catastrophic effects around the globe have resulted in more than 6 million deaths (2). COVID-19 mortality was 6.3% in Brazil (3) and 3.6% in China (4). The WHO African Region (WHO AFR) accounted for 82.7% of cases and 76.2% of deaths around the globe. As of February 24, 2023, 10.8 million COVID-19 cases were reported in Africa, with 228,738 deaths (CFR: 2.1%) and 9.8 million recoveries (93.8%) (5, 6). The incidence rate of mortality was 0.203 per 1,000 persons per day in the WHO African region (7). In studies of sub-Saharan Africa, the pooled prevalence of mortality in COVID-19 patients was 4.8% (8) and 2.4% (9). In Kenya, 14% of patients with COVID-19 died (10). In Ethiopia, the case fatality rate of COVID-19 ranges between 1 and 20% (11) while the incidence of COVID-19 mortality was 9.13 per 1,000 person-days in Ethiopia (12). The effectiveness of COVID-19 mitigation and patient clinical outcomes are influenced by the economy and medical resources. A study’s findings revealed that the COVID-19 pandemic is likely to get worse in developing nations, making low-income countries more vulnerable if it is not controlled (13). Cultural norms affect COVID-19-related mortality (14). Individualism societies were favorably correlated with COVID-19 prevalence, mortality, and case fatality rates; in contrast, collectivism societies were adversely correlated with these characteristics. This correlation between individualism and the severity of the virus problem may be explained by the fact that social non-cooperativeness in individualistic societies decreases the effectiveness of non-pharmaceutical treatments intended to alleviate the virus problem (15, 16). A high mortality rate from COVID-19 has been reported in patients with comorbidities (17–19). The risk of COVID-19 mortality was increased for those with advanced age, male gender, hypertension, diabetes mellitus, cardiovascular disease, and cancer (20–24). Patients with acute respiratory distress syndrome, which is a potentially fatal disease, were more likely to experience severe COVID-19 morbidity and mortality (25). Hand washing, social distancing, and quarantine are the key strategies for controlling the spread of the COVID-19 disease in society (26). Despite the extensive implementation of pandemic prevention and control measures in Ethiopia with the active participation of policymakers’ leadership, healthcare facilities encountered shortages of emergency care medical supplies, ventilators, medical equipment, oxygen supplies, and well-ventilated isolation rooms (27). Various studies reported that the COVID-19 pandemic in low-income countries would continue to worsen, with potentially fatal results.

There was a systematic review of the incidence rate of COVID-19 mortality and its predictors in Ethiopia, published in 2023 (12). This study only included studies with incidence rates without considering prevalence studies, which could result in the study’s power being inadequate to estimate the mortality rate of COVID-19, as only a limited number of studies with an incidence rate were used to determine the mortality rate of COVID-19. However, the current systematic review conducted to estimate the pooled mortality rate of COVID-19 and its predictors increased the power of the study by including both prevalence and incidence studies of COVID-19 mortality compared to the previous study. In this context, a systematic review and meta-analysis was conducted to estimate the accurate burden of the COVID-19 mortality rate and its associated risk factors in Ethiopia since the current study improved its power.

### Objectives

The first objective of this review was to determine the pooled prevalence of COVID-19 mortality among coronavirus 2-infected patients in Ethiopia. The second objective was to determine the pooled incidence rate of mortality among COVID-19 patients, which can be used to measure the risk of death from COVID-19 disease over some specific period of time that can aid in recognizing whether the condition is increasing, decreasing, or remaining static. Finally, this systematic review and meta-analysis identified risk factors associated with the COVID-19 mortality rate in Ethiopia. To prevent COVID-19 mortality, determining the magnitude of COVID-19 mortality and a thorough exploration and understanding of its risk factors were inputs for policymakers and programmers to establish prevention strategies that helped control the pandemic’s adverse health effects or minimize its risk factors.

### Review question

1. What is the pooled prevalence of mortality among COVID-19 patients in Ethiopia?
2. What is the pooled incidence rate of mortality among COVID-19 patients in Ethiopia?
3. What are the contributing factors to the mortality among COVID-19 patients in Ethiopia?

## Methods

### Protocol and registration

The Preferred Reporting Items for Systematic Reviews and Meta-Analyses guidelines were followed in this systematic review and meta-analysis (28) (Supplementary material S1). The protocol has been published in PROSPERO with ID CRD42020165740.

### Searching strategies

Two authors (TGW and JAM) ran the search strategy across pertinent databases using an advanced search strategy that was designed, constructed with Boolean operators, and matched to accessible and available databases. Using the main databases of PubMed, CINAHL, Google Scholar, and African Journals Online, all pertinent published articles were pulled up. The following keywords were used to search all electronic databases: mortality, COVID-19, and Ethiopia. The first search strategies were conducted from May 19, 2023, to May 21, 2023, and then updated from January 22, 2024, to January 27, 2024. The comprehensive search strategies were found in the Supplementary material S2.

### Selection of studies for inclusion in the review

Articles found through various database searches were combined and exported to Endnote X8.1 software. Duplicate articles were eliminated using Endnote X8.1 software. TGW and JAM independently screened the selected articles for relevance to the review objective using their titles and abstracts. Then, the full texts of all articles deemed relevant in the initial screening were assessed independently by two reviewers (TGW and JAM) for their eligibility to be included in the final analysis. If there was disagreement between two authors, the third expert’s colleague reached a consensus.

### Outcome measurement

In this study, the mortality rate was determined as the percentage of COVID-19 cases having a death outcome. Two parameters were necessary to calculate the COVID-19 mortality rate: the total number of confirmed COVID-19 patients and the number of deaths from COVID-19 cases. Based on this, the mortality rate was estimated by dividing the number of deaths from COVID-19 cases by the total number of confirmed COVID-19 patients (sample size).

### Eligible criteria

The PICO technique, which primarily uses condition, context, and population (CoCoPop) questions, was employed by the authors to determine the inclusion and exclusion criteria for this systematic review and meta-analysis. Study subjects must be COVID-19 patients with laboratory confirmation. Studies must record their outcomes as COVID-19 mortality or death, or the availability of enough data to determine the mortality (the total number of confirmed COVID-19 patients along with the number of COVID-19 death cases). Studies must be published in English, and there are no restrictions on the inclusion of studies based on publication type. A study must be conducted in Ethiopia. Studies with case reports, review articles, commentary, letters to the editor, and studies without full text were excluded. Studies with poor methodology or those that failed to clearly report mortality or death as the outcome or its risk factors were also excluded.

### Data extraction

TGW and JAM independently extracted all necessary data from the original articles. Disagreements were resolved by consensus. The data were extracted as a summary table in Microsoft Excel using a standardized data extraction format. For each included study, the first author’s name, the publication year, the study period, region, study design, the total number of confirmed COVID-19 patients, and the number of COVID-19-dead cases (Supplementary material S3).

### Appraisal of the included studies’ quality

The Preferred Reporting Items for Systematic Reviews and Meta-Analyses (PRISMA) checklist was used to verify scientific validity. The quality of each included study was assessed independently by two investigators (TGW and JAM) using a standardized set of criteria from the Newcastle-Ottawa Scale (NOS) (29). Any disagreements were resolved at the invitation of a third expert. Three domain categories were included in the Newcastle-Ottawa Quality Assessment Form for Cohort Studies: selection (representativeness of the exposed cohort, selection of the non-exposed cohort, and ascertainment of exposure and outcome of interest not present at the start of the study), comparability, and outcome (assessment of outcome, long enough follow-up for outcome occurrence, and adequate follow-up of cohorts). The quality scores of the included studies were classified as low quality (<50%), medium quality (50–69%), and high quality (≥70%). Finally, articles scoring at least half (50%) of the total score were considered of good quality and included in the meta-analysis.

### Statistical analysis

All the relevant data was extracted from the included studies using a Microsoft Excel spreadsheet, which was then imported to STATA software version 15 for further analysis (30). A random-effects model was applied due to the expected heterogeneity (31). I^2^ is used to estimate the percentage of overall variation across studies due to heterogeneity. I^2^ equals 25, 50, and 75%, which were regarded as low, moderate, and high levels of heterogeneity, respectively (32). When heterogeneity was greater than 50% (based on the I^2^ statistic), the random-effects model was applied. Subgroup analyses were carried out to identify potential heterogeneity moderators in cases of substantial heterogeneity (33). A funnel plot and the Egger regression test were employed to assess publication bias (34, 35). The trim-and-fill technique was used to correct publication bias (36). Sensitivity analysis was carried out by omitting each study and computing the *p* values of the studies that remained (37). The pooled effect size was also computed by the metan command using each factor’s adjusted odd ratio (AOR) to determine the association between mortality and its risk factors. Tables and forest plots with a 95% CI were then used to summarize the findings of each selected article.

## Results

### Search results

**Figure 1:**
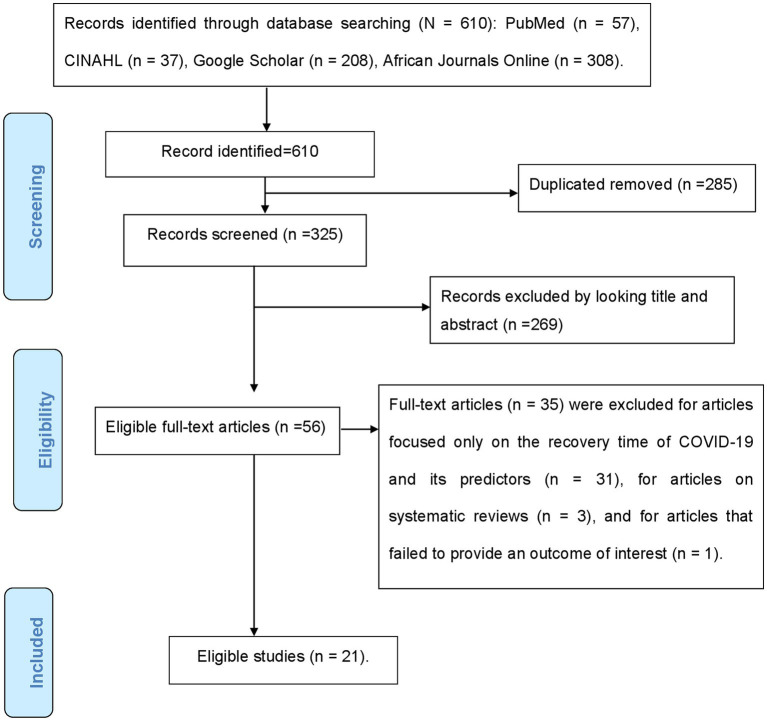
PRISMA flow chart for the selection of studies for systematic review.

Electronic databases brought up 610 articles on COVID-19 mortality during the initial search. Two hundred eighty-five were removed as a result of duplication, and 269 were excluded after title and abstract screening because they did not relate to the study’s objective. Fifty-six articles were reviewed for full text; 35 of them were excluded for a number of reasons, including the fact that 31 of the studies focused only on the recovery time of COVID-19 and its predictors, three of them were systematic reviews, and one did not provide an outcome of interest. The final analysis included 21 studies that met the eligibility criteria. The specific screening procedures are depicted in a PRISMA flow chart (Figure 1).

### Characteristics of eligible studies

A total of 21 studies with 42,307 study participants were included in the final analysis. Out of a total of 21 eligible studies, one was a prospective cohort study (38), and the remaining 20 were retrospective cohort studies. Ten studies reported both the magnitude of COVID-19 mortality and its determinants; 11 studies reported only the magnitude of mortality. In this study, there were six regions and one administrative city (Addis Ababa). Most studies were conducted in the Oromia region (38–44). In the Amhara region (45–47) and the Southern Nation Nationality of People (SNNP) (48–51), three and four studies were conducted, respectively. Addis Ababa (52–54) and Harari (55, 56), had three and two studies, respectively. Tigray (57) and Benshangul Gumz (58) each only had one study. The smallest and largest sample sizes observed in the Tigray (57) and Amhara regions (45) were 139 and 28,533, respectively. The lowest magnitude of COVID-19 mortality (1.2%) was recorded in the Oromia region (41), while the highest magnitude (63.3%) was recorded in Addis Ababa (53). The maximum and minimum incidence rates of COVID-19 mortality were 56.7 per 1,000 persons per day (53) and 4.7 per 1,000 persons per day (46), respectively. The majority of studies (61.9%) were conducted between 2020 and 2021 (Table 1).

**Table 1:** Characteristics of eligible studies (*n* = 21).

ID	Author, publication year	Region	Study design	Study period	Sample size (*N*)	Cases	*P* (%)	IR per PD
1	Kaso et al. (39), 2022	Oromia	Retrospective	2020–2021	308	50	16.2%	–
2	Kaso et al. (40), 2022	Oromia	Retrospective	2020–2021	422	47	11.13%	6.35 per 1,000
3	Birhanu et al. (55), 2022	Harari	Retrospective	2020–2021	355	96	27.04%	–
4	Dessie et al. (45), 2022	Amhara	Retrospective	2020–2021	28,533	2,873	10.07%	11.78 per 1,000
5	Mengist et al. (46), 2022	Amhara	Retrospective	2020–2021	522	29	5.56%	4.7 per 1,000
6	Tamiru et al. (47), 2023	Amhara	Retrospective	2020–2021	452	37	8.2%	–
7	Getahun et al. (54), 2023	Addis Ababa	Retrospective	2021	393	32	8.1%	–
8	Habtewold et al. (38), 2022	Oromia	Prospective	2021	852	97	11.4%	9.9 per 1,000
9	Gudina et al. (41), 2021	Oromia	Retrospective	2020	4,398	52	1.2%	–
10	Kebede et al. (58), 2022	Benishangul Gumuz	Retrospective	2020	288	50	17.4%	1.8 per 100
11	Ayana et al. (56), 2021	Harari	Retrospective	2020–2021	531	101	19.02%	16.2 per 1,000
12	Churiso et al. (48), 2022	SNNP	Retrospective	2020–2021	220	49	22.3%	–
13	Nega et al. (53), 2022	Addis Ababa	Retrospective	2020–2021	496	314	63.3%	56.7 per 1,000
14	Abebe et al. (57), 2022	Tigray	Retrospective	2020	139	56	40.3%	–
15	Lemma Tirore et al. (49), 2022	SNNP	Retrospective	2020–2021	845	70	8.3%	–
16	Misganaw et al. (50), 2023	SNNP	Retrospective	2020–2021	1,032	128	12.4%	–
17	Tsegaye et al. (42), 2022	Oromia	Retrospective	2020–2022	300	13	4.3%	–
18	Tolossa et al. (43), 2021	Oromia	Retrospective	2020	263	15	5.7%	–
19	Tolossa et al. (44), 2022	Oromia	Retrospective	2020–2021	318	51	16.04%	14.1 per 1,000
20	Atamenta et al. (52), 2023	Addis Ababa	Retrospective	2020	602	87	14.5%	10.7 per 1,000
21	Fantaw et al. (51), 2023	SNNP	Retrospective	2020–2022	1,038	181	17.4%	–

PD, person day; IR, incidence rate; Dash (−) indicates the data is not available or has not been reported. SNNP, Southern Nation Nationality of People; P, prevalence.

### Quality assessment of included studies

Two authors independently assessed the quality of the included papers using New Castle-Ottawa for cohort studies. For the prospective cohort study, a total of nine scores were used. A total of seven scores were used for retrospective cohort studies since two quality assessments—length of follow-up and adequate (completeness) of follow-up—were ignored because they are not applicable for retrospective cohorts. There was only one prospective cohort study that scored a perfect nine (100%). In a retrospective cohort study, 10 included studies scored 100%, while the other ten scored 71.4%. In general, all the included studies had high quality. The two authors’ mutually agreed-upon assessment of the quality of each included study is presented in the Supplementary material S4.

### The pooled prevalence of COVID-19 mortality

**Figure 2:**
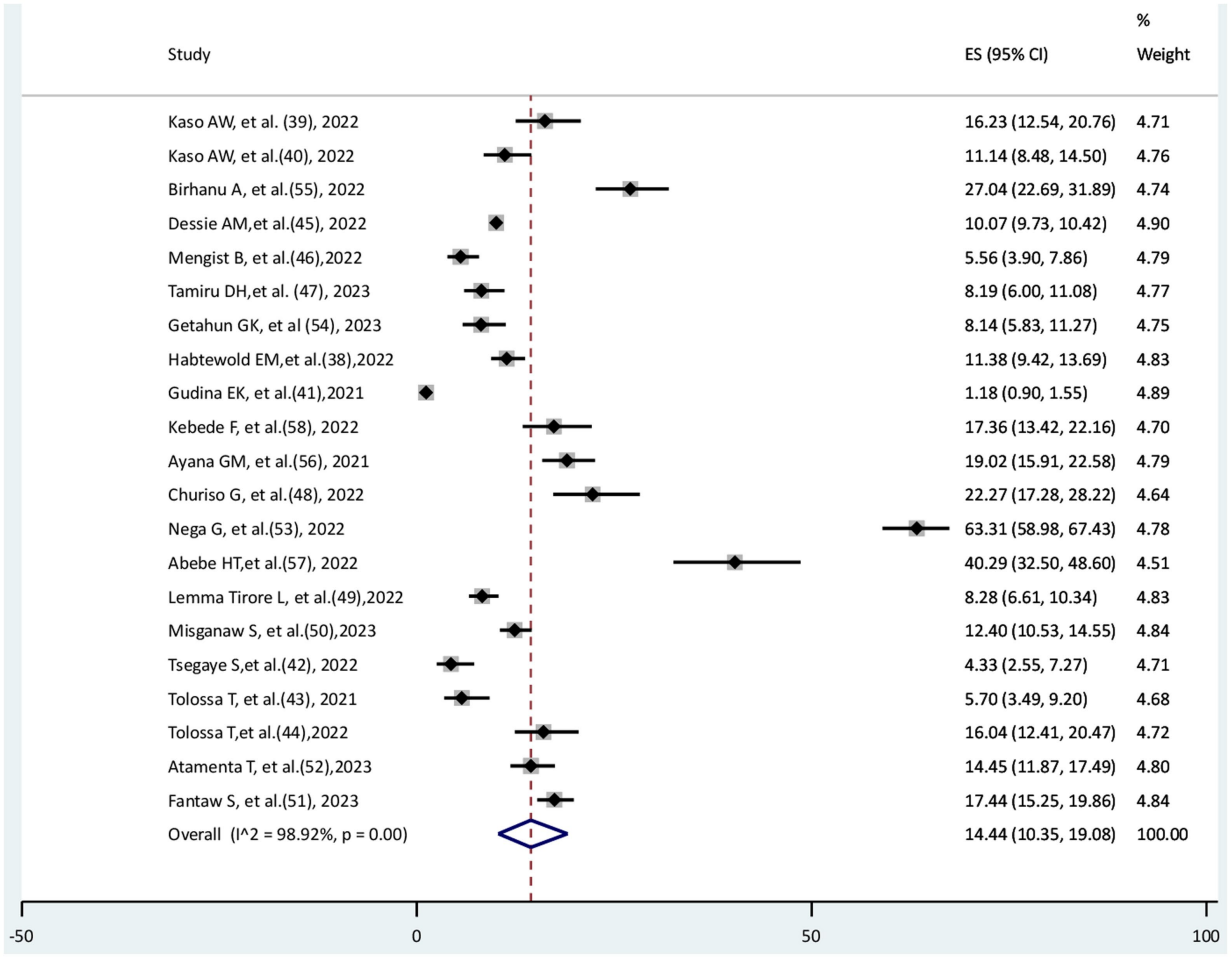
The pooled prevalence of COVlD-19 mortality.

In this systematic review and meta-analysis, 21 eligible studies were included to determine the pooled prevalence of COVID-19 mortality. A total of 42,307 COVID-19 patients were included; of them, 4,428 died. The pooled prevalence of COVID-19 mortality was 14.44% (95% CI: 10.35–19.08%), with high significant heterogeneity (I^2^ = 98.92%, *p* < 0.001) (Figure 2).

### The pooled incidence rate of COVID-19 mortality

**Figure 3:**
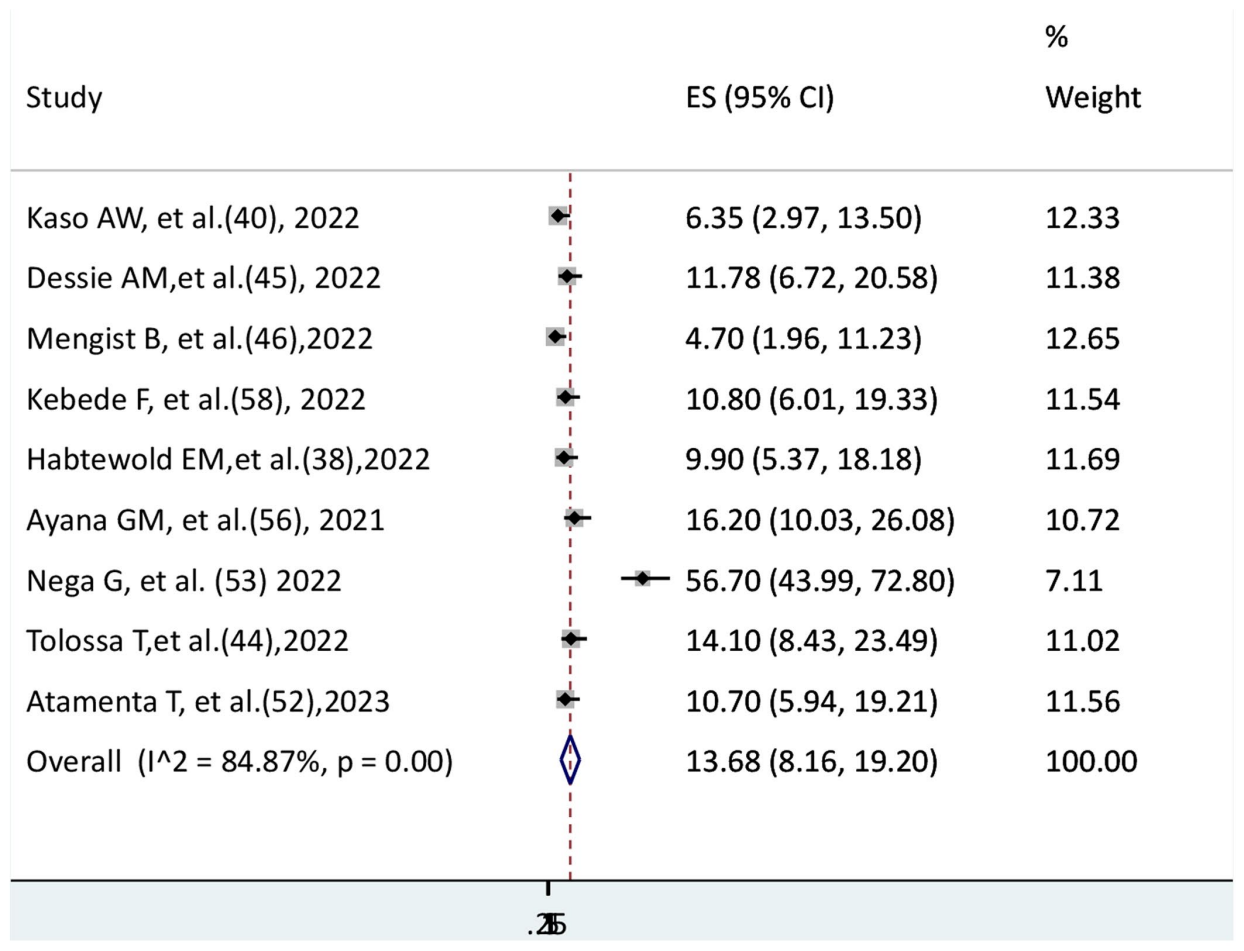
The pooled incidence rate of COVID-19 mortality per 1000 persons per day.

The incidence rate of COVID-19 mortality was available in nine studies; the other studies could not report the incidence rate. Thus, these nine studies were eligible to estimate the pooled incidence rate of COVID-19 mortality among the 21 included studies. The pooled incidence rate of COVID-19 mortality was reported as 1,000 persons per day. In this systematic review and meta-analysis, the pooled incidence rate of COVID-19 mortality was 13.68 (95% CI: 8.16–19.2) per 1,000 persons per day. There is significant heterogeneity among the included studies (I^2^ = 84.87%, *p* < 0.001) (Figure 3).

### Subgroup analysis of the prevalence of COVID-19 mortality

The highest prevalence of COVID-19 mortality rate was observed in the Tigray region (40.3, 95% CI: 32.5–48.6%), followed by Addis Ababa (25.9, 95% CI: 2.3–62.6%), with the absence of heterogeneity. The highest significant level of heterogeneity occurred in the Oromia region (I^2^ = 98.4%, *p* < 0.001) and southern nation nationality of peoples (SNNP) (I^2^ = 93.9%, *p* < 0.001). The prevalence of COVID-19 mortality rate was found to be 14.6% (95% CI: 10.3–19.5%) in retrospective studies, with significant heterogeneity (I^2^ = 99%, *p* < 0.001). The maximum prevalence of COVID-19 mortality (16.8, 95% CI: 11.1–23.5%) was observed in studies conducted between 2020 and 2021, with significant heterogeneity (I^2^ = 98.7%, *p* < 0.001). In sample sizes of less than 422, the prevalence of COVID-19 mortality was 16.1% (95% CI: 10–23.2%), with high heterogeneity (I^2^ = 95.4%, *p* < 0.001) (Table 2).

**Table 2:** Subgroup analysis of COVID-19 mortality in Ethiopia (*n* = 21).

Variables	Categories	Included study	Sample size	Mortality rate (95%CI)	Heterogeneity (I^2^, *p*-value)
Region	Oromia	7	6,861	8.5% (3.18–15.9%)	98.4%, *p* < 0.001
	Amhara	3	29,507	8% (5.4–11.1%)	–
	SNNP	4	3,135	14.5% (9.7–20.04%)	93.9%, *p* < 0.001
	Addis Ababa	3	1,491	25.9% (2.3–62.6%)	–
	Harari	2	886	22.1% (19.4–24.9%)	–
	Tigray	1	139	40.3% (32.5–48.6%)	–
	Benishangul Gumuz	1	288	17.4% (13.4–22.2%)	–
Study design	Retrospective	20	41,455	14.6% (10.3–19.5%)	99%, *p* < 0.001
	Prospective	1	852	11.4% (9.4–13.7%)	–
Study period	2020	5	5,690	13.0% (3.04–28.3%)	99.04%, *p* < 0.001
	2020–2021	12	34,034	16.8% (11.1–23.5%)	98.7%, *p* < 0.001
	2021	2	1,245	10.3% (8.7–12.1%)	–
	2020–2022	2	1,338	13.8% (12–15.8%)	–
Sample size	< 422	9	2,584	16.1% (10–23.2%)	95.4%, *p* < 0.001
	≥ 422	12	39,723	13.3% (8.2–19.5%)	99.3%, *p* < 0.001

SNNP, Southern Nation Nationality of People.

### Publication bias

**Figure 4:**
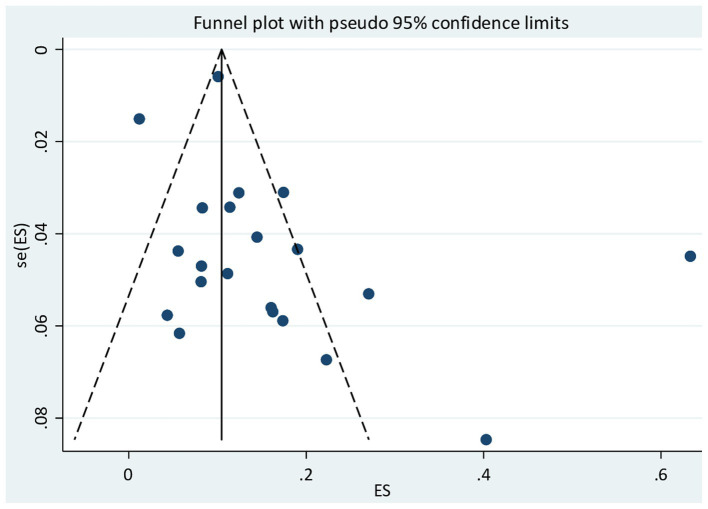
Funnel plot for publication bias for the pooled prevalence of COVID-19 morality.

In this meta-analysis, Egger’s test was employed to check for the existence of publication bias among the 21 included studies. Egger’s test for a regression intercept obtained a *p*-value of 0.102, demonstrating no evidence of publication bias. There is possible evidence of publication bias in the funnel plot for Figure 4.

### Sensitivity analysis

**Figure 5:**
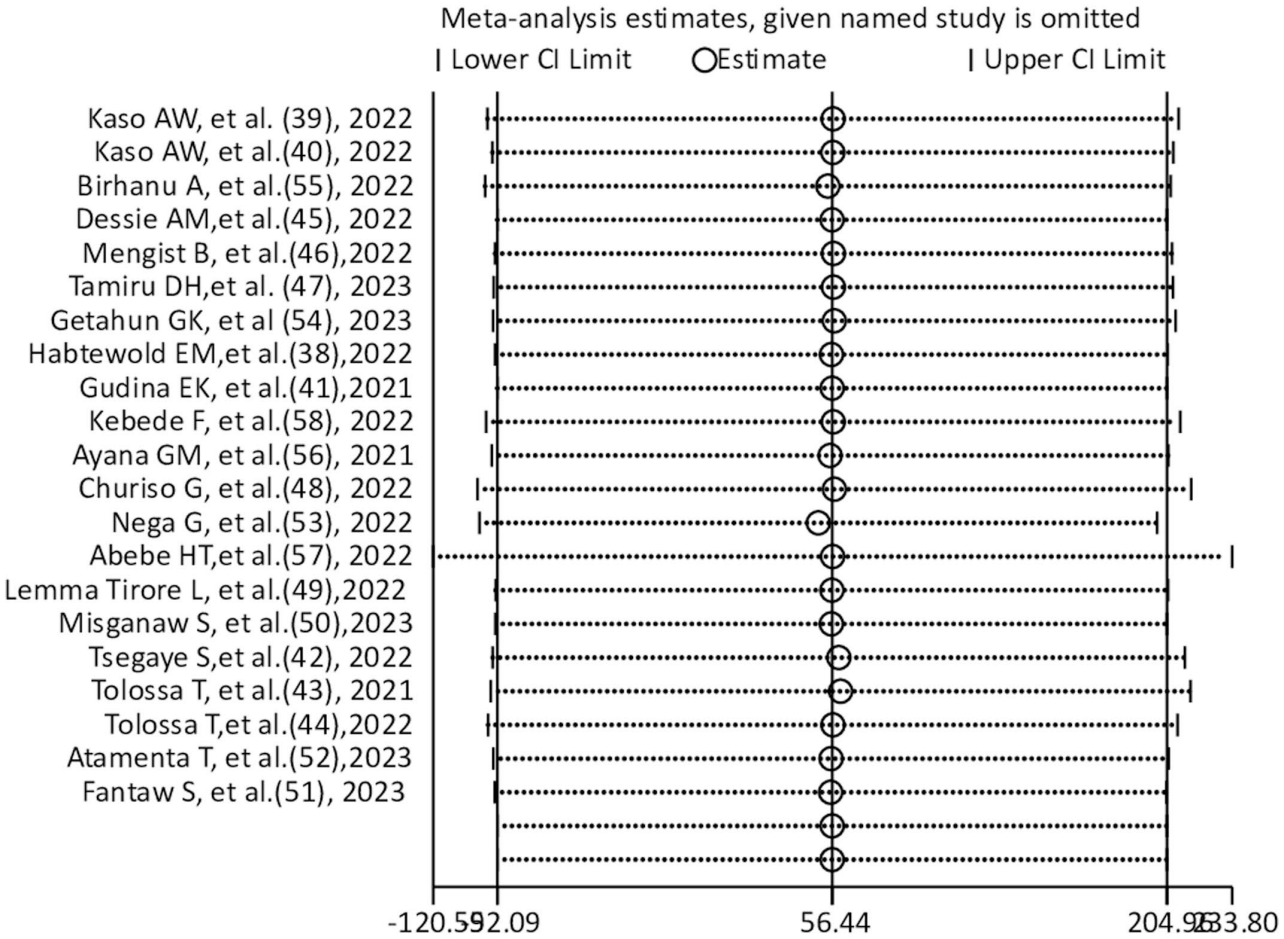
Sensitivity analysis for pooled prevalence of COVID-19 morality.

A sensitivity analysis was carried out to see if any small study effects influenced the pooled effect size among included studies (*n* = 21). According to the leave-one-out sensitivity analysis, there were not any detectable differences (Figure 5). The current meta-analysis’s findings appear to be fairly consistent.

### Determinants of COVID-19 mortality rate

For this systematic review, comorbidity, hypertension, cardiovascular disease, and kidney disease were eligible factors to conduct a meta-analysis. The risk of dying for patients with comorbidity and cardiovascular disease was significantly higher than that for those without conditions. However, those patients with kidney disease and hypertension did not have a significant difference in the COVID-19 mortality rate compared with their counterparts.

### The risk of COVID-19 mortality among patients with comorbidity

**Figure 6:**
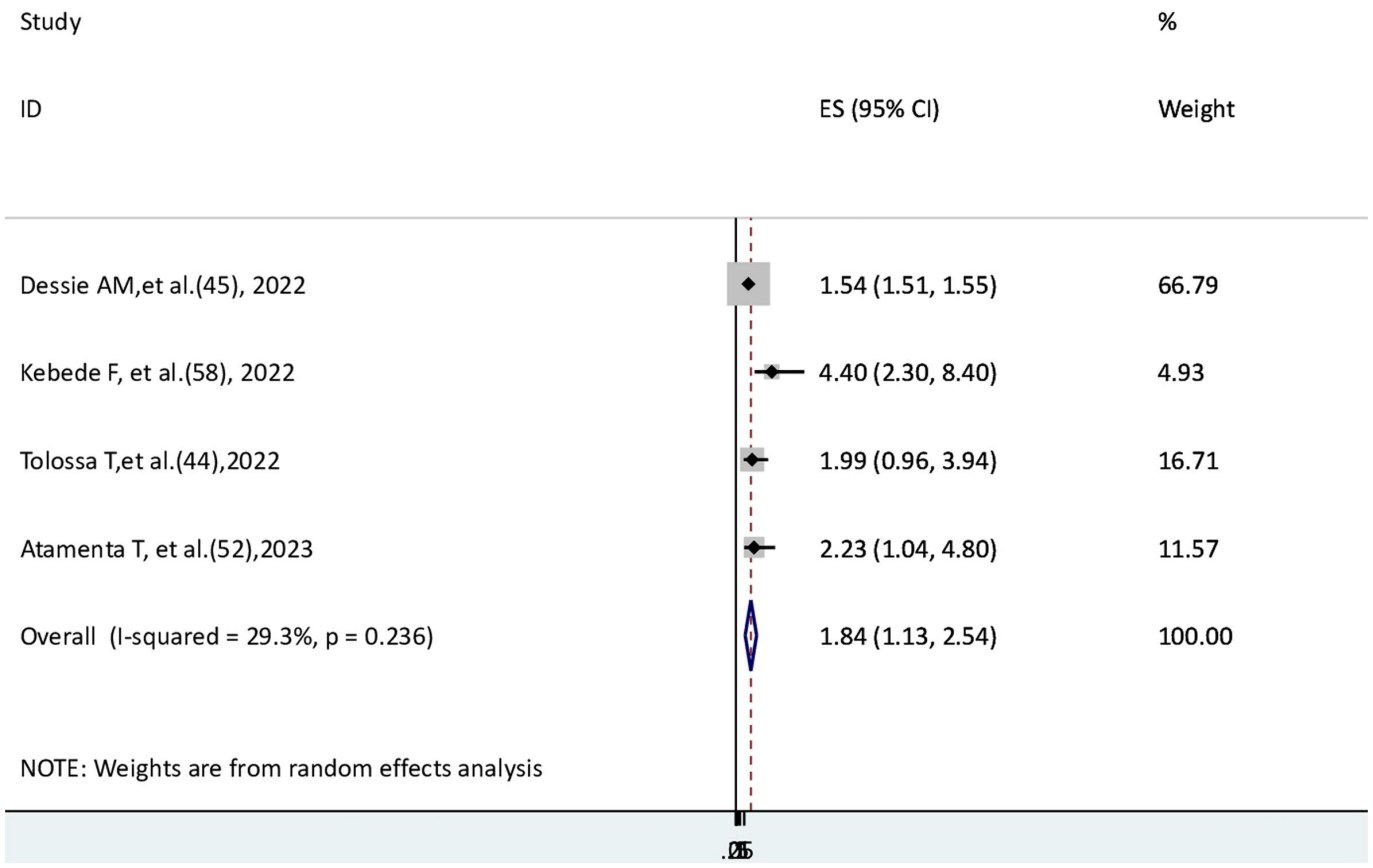
The association between COVID-19 morality and comorbidity.

To check the risk of COVID-19 mortality for patients with comorbidity, four studies (44, 45, 52, 58) were found; three of them (45, 52, 58) revealed that the risk of COVID-19 mortality was statistically significant, and one (44) did not have a significant association. This meta-analysis showed that the hazard of death from COVID-19 disease for patients with comorbidity was 1.84 times higher than that of patients without comorbidity (AHR = 1.84, 95% CI: 1.13–2.54), with moderately insignificant heterogeneity (I^2^ = 29.3%, *p* = 0.236) (Figure 6).

### The risk of COVID-19 mortality for patients with hypertension

**Figure 7:**
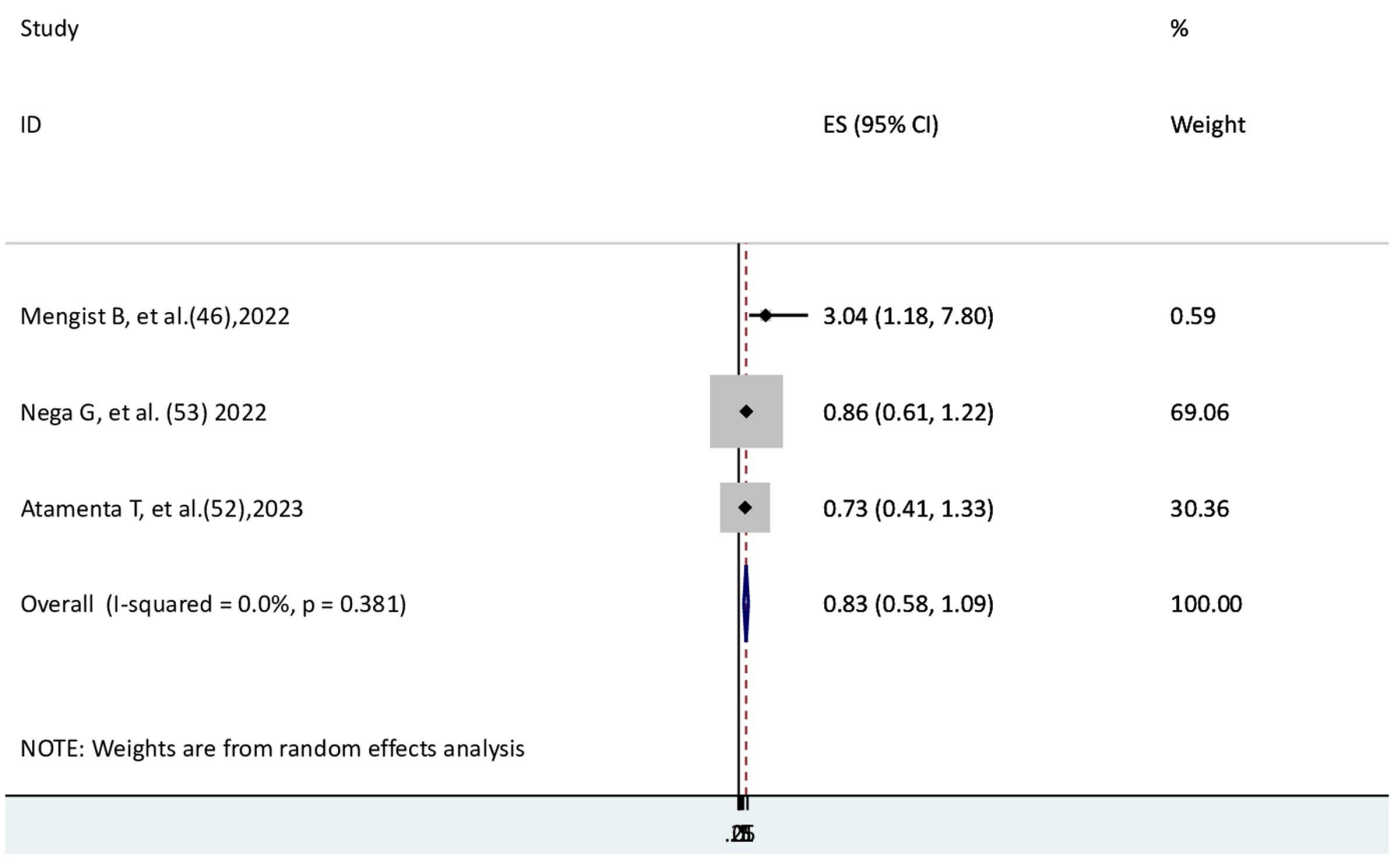
The association between COVID-19 morality and hypertension.

Three studies (46, 52, 53) were used to examine the relationship between hypertension and COVID-19 mortality. One study (46) showed a significant association, whereas the other two studies (52, 53) revealed no significant association. In the current meta-analysis, the risk of COVID-19 mortality did not differ significantly between patients with and without hypertension (AHR = 0.83, 95% CI: 0.58–1.09) in the absence of heterogeneity (I^2^ = 0.0) (Figure 7).

### The risk of COVID-19 mortality for patients with cardiovascular disease

**Figure 8:**
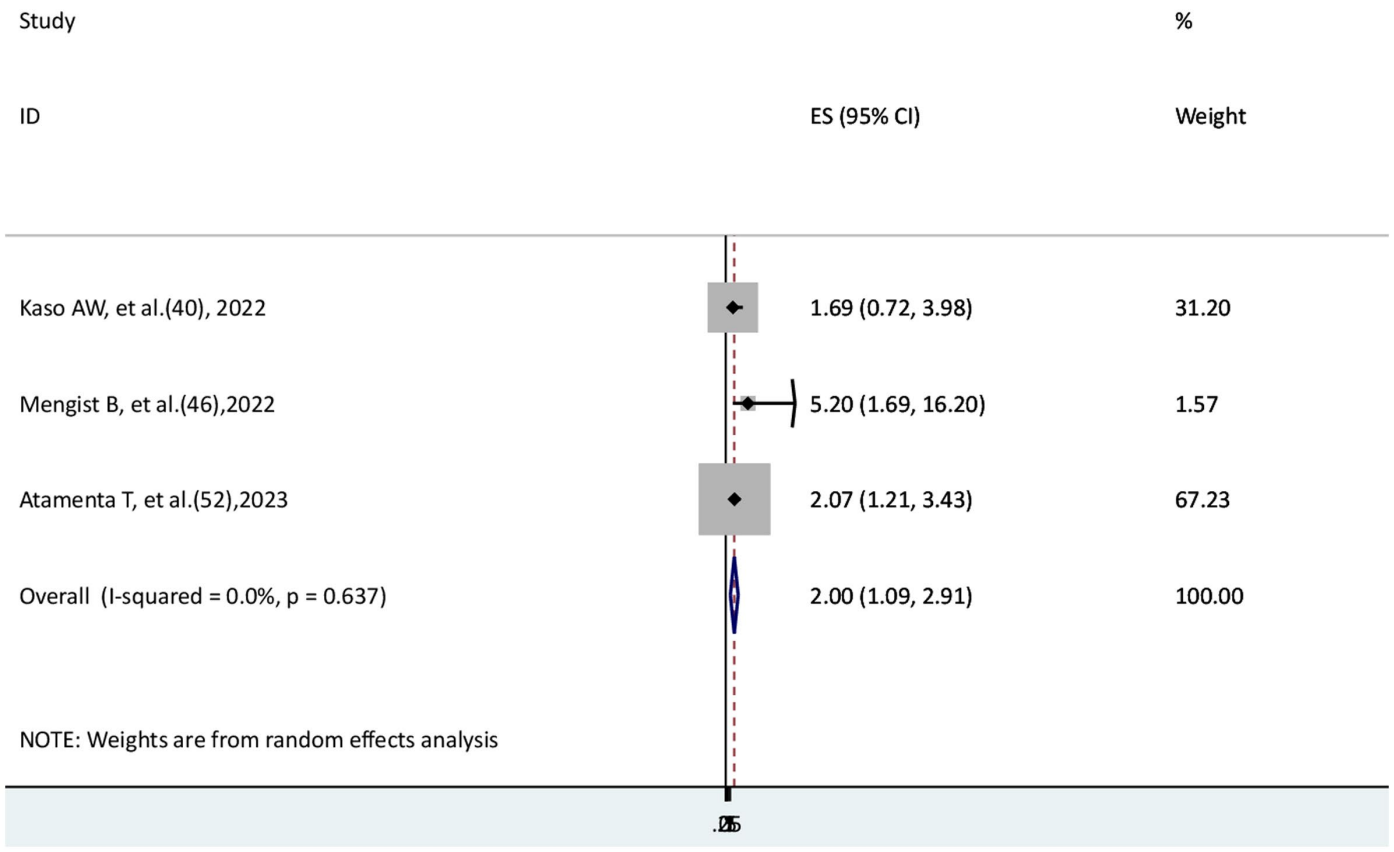
The association between COVID-19 morality and cardiovascular disease.

The risk of COVID-19 mortality rate for patients with cardiovascular disease was examined in a total of three studies (40, 46, 52). Two studies (46, 52) found a significant association, while another study (40) found no significant association. The findings of this systematic review and meta-analysis showed that the hazard of mortality from COVID-19 disease for patients with cardiovascular disease was two times higher than that of those who did not (AHR = 2, 95% CI: 1.09–2.91) with no heterogeneity (I^2^ = 0.0) (Figure 8).

### The risk of COVID-19 mortality for kidney disease patients

**Figure 9:**
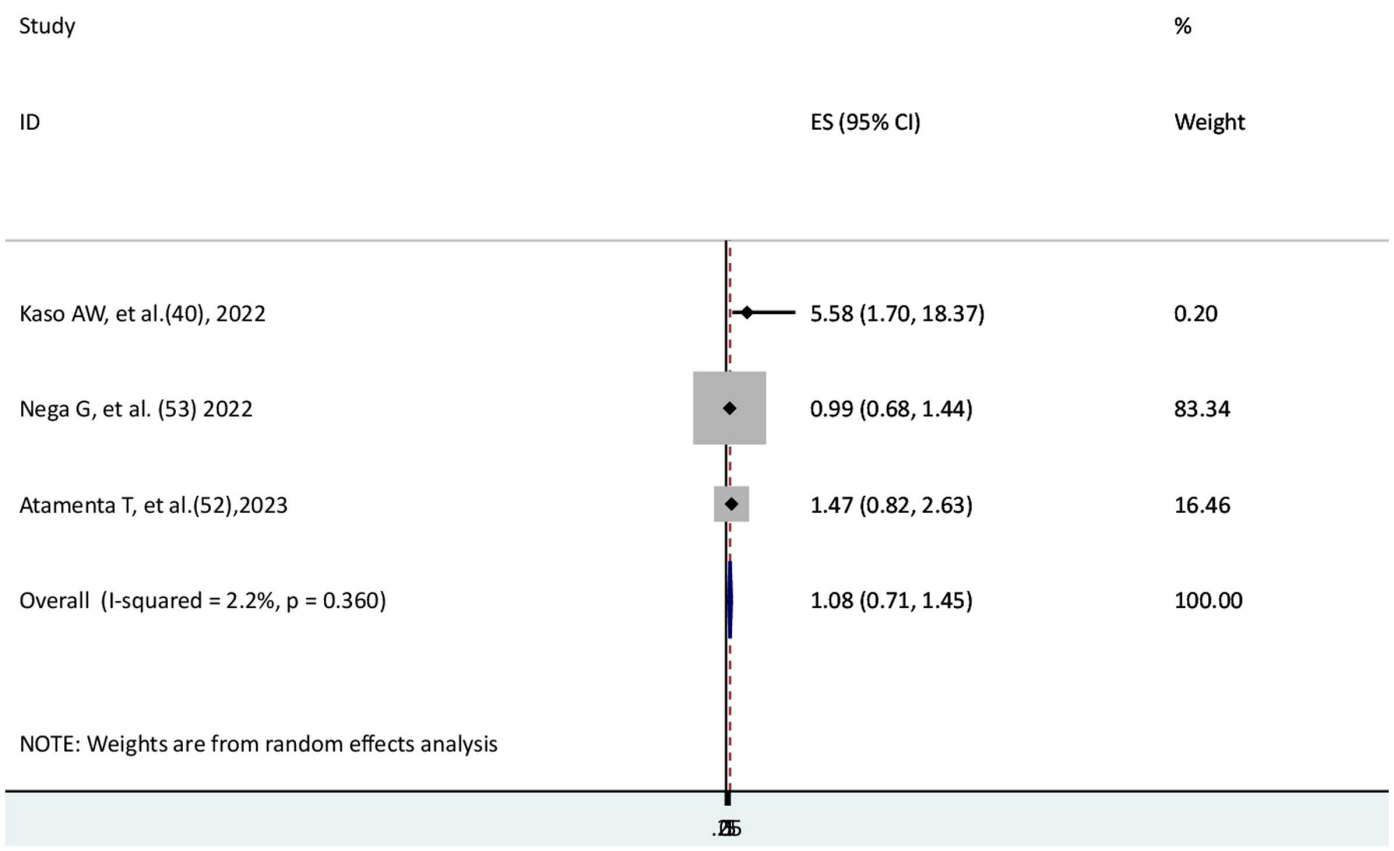
The association between COVID-19 morality and kidney disease.

Three studies (40, 52, 53) were used to assess the risk of COVID-19 mortality for patients with kidney disease. One study (40) indicated a significant relationship, but two studies (52, 53) reported no significant difference in COVID-19 mortality between patients with and without kidney disease. The result of the meta-analysis revealed that the risk of mortality from COVID-19 disease did not significantly differ between those patients with and without kidney disease (AHR = 1.08; 95% CI: 0.71–1.45). Low heterogeneity was reported (I^2^ = 2.2%, *p* = 0.36) (Figure 9).

## Discussion

In order to limit the COVID-19 pandemic and its adverse outcomes on health, such as the severity of the illness and case fatality rate, evidence-based studies and coordinated preparedness and response efforts at national, regional, and global levels are required. This study serves to determine the magnitude of COVID-19 deaths and explore its risk factors in Ethiopia, aiming to establish preventive strategies. In this meta-analysis, the pooled prevalence of COVID-19 mortality was 14.66% (95% CI: 10.35–19.08%). This magnitude of the finding is significantly higher than studies conducted in Brazil (3) and China (4). This variation may be explained by the disparities in medical resources (13), including shortages of emergency care supplies, ventilators, equipment, oxygen supply, and well-ventilated isolation rooms in Ethiopia (27). Moreover, the current pooled mortality rate of COVID-19 is substantially higher than that of the WHO Africa region (5, 6), and studies conducted in sub-Saharan Africa (8, 9). According to the global report, COVID-19 mortality rates in Ethiopia are relatively low, which is probably underreported. However, it is comparable with studies conducted in Kenya (10) and Ethiopia (11). The difference may be related to a difference in cultural norms (14), i.e., countries with more individualistic societies may be affected more by COVID-19 mortality and case fatalities. These facts are associated with the fact that social lack of cooperation in individualistic societies decreases the effectiveness of non-pharmacological treatments intended to alleviate the disease (15, 16). In this meta-analysis, the pooled incidence rate of COVID-19 mortality was 13.68 (95% CI: 8.2–19.2) per 1,000 persons per day. This finding is much higher than the WHO Africa region, which reported 0.0203 per 1,000 persons per day (7), and a systematic study in Ethiopia that reported 9.13 per 1,000 person-days (12). This indicates that COVID-19 mortality is still rising. This can be a result of a shortage of late detection of cases, ventilators, oxygen, and other medical supplies and equipment in Ethiopia (27). Another explanation for the difference in mortality might be that the majority of the study participants were in the older age group (20–24) and had comorbidities (17–19) in Ethiopia. Another factor contributing to the difference between the incidence rate of COVID-19 mortality in the current study and a previous systematic review and meta-analysis study (12) was the inclusion of additional studies in the current systematic review and meta-analysis study, which increased the study’s sample size and helped in detecting differences more accurately. The findings of this study suggest that the Ethiopian Ministry of Health should give more attention to reducing the mortality of COVID-19 diseases through early detection of the disease (before severe complications) and appropriate management by providing adequate treatments and assisted instruments. The COVID-19 mortality rate was highest in the Tigray region (40.3%), followed by Addis Ababa (25.9%), while Amhara (8%) and Oromia (8.5%) had the lowest COVID-19 mortality in comparable percentages. This variation may be due to the fact that studies in regions with the highest COVID-19 mortality rates were done among critically ill COVID-19 patients, whose risk of death is increased. The sample size is the other possible variation for the differences in outcome occurrence because, in most of the region, a small number of studies with varying sample sizes were undertaken, which in turn indicates that the sample size is insufficient to detect the outcome. The COVID-19 mortality rate was slightly higher in retrospective studies with significant heterogeneity compared to a prospective study without heterogeneity. The variation may be due to the fact that the lack of heterogeneity in a prospective study implies that the outcome may not be detected precisely, possibly as a result of chance (small sample size). The COVID-19 mortality rate was 13% in 2020, 16.8% in 2020–2021, 10.3% in 2021, and 13.3% in 2020–2022. This indicates that from the early pandemic era (2020) to 2020–2021, the death rate rose, and it decreased from 2021 to 2022. The reduction in the COVID-19 case fatality rate could be attributed to the COVID-19 pandemic’s decline in 2021 as a result of the boosting of the COVID-19 vaccine and therapies. The decrease in mortality may also be attributed to the active implementation of pandemic prevention and control policies in Ethiopia (27), such as hand washing, social distance, and quarantine, which served to reduce disease transmission (26). The pooled prevalence of COVID-19 mortality was 13.3% in sample sizes greater than or equal to 422, while it was 16.1% in sample sizes less than 422. The existence of study participants with various comorbidities in these study groups could be the reason for this discrepancy.

The hazard of death from COVID-19 was greater for patients with comorbidity than for those without comorbidity. This evidence is consistent with the previous studies’ findings (17–19). Patients with cardiovascular disease had a higher risk of COVID-19 death than patients without this condition. These findings are in line with those from past studies (20–24). The increased risk of COVID-19 death for patients with comorbidities, including cardiovascular disease, shows the epidemiological effect of these diseases on people with chronic diseases. The reasons may be that the numerous pathophysiologic effects of chronic diseases, including cardiovascular disease, are significant for the outcomes of an infectious disease. For instance, inflammatory cytokines like IL-1β and TNFα may be released as a result of autoimmunity, which contributes to a chronic inflammatory state. Patients with kidney disease and hypertension did not differ significantly from those without kidney disease and hypertension in terms of their risk of COVID-19 mortality. The absence of a statistically significant difference may be attributable to the studies’ inclusion of a small number of patients with kidney disease and hypertension, which made it challenging to detect differences due to the effect of chance.

The limitation of this study is that it did not include all regions found in Ethiopia. The other limitation of this study is that the majority of the included studies were retrospective, which relies on records with limited variables that affect data validity and makes it challenging to control all confounding factors. There was a high level of heterogeneity across studies. There are heterogeneities even after subgroup analysis in studies conducted in the Oromia region and southern nation nationality of people, in studies using retrospective study designs, in the study period (2020 and 2020–2021), and in study sample size (sample size<422 vs. ≥422). The occurrence of high heterogeneity may be caused by variations in the socio-demographic characteristics of study participants, the presence of comorbidity among study participants, and studies conducted on critically ill patients. Only one or a few studies were carried out across the majority of the region, which implies the sample size is inadequate to detect the outcome. Even though the study had shortcomings, it actually contributed to the development of evidence-based healthcare that helped reduce COVID-19 mortality.

## Conclusion

Substantial numbers of COVID-19 patients died in Ethiopia. The hazard of COVID-19 mortality for patients with comorbidities and cardiovascular disease was higher than that of patients without these diseases. For these COVID-19 mortality-prone patients, intensive surveillance, patient monitoring, and early medical intervention should be required. The distribution of the COVID-19 vaccination should be boosted, especially for people with comorbid conditions like cardiovascular disease.

## Data availability statement

The original contributions presented in the study are included in the article/Supplementary material, further inquiries can be directed to the corresponding author.

## Author contributions

TGW: Conceptualization, Data curation, Formal analysis, Funding acquisition, Investigation, Methodology, Project administration, Resources, Software, Supervision, Validation, Visualization, Writing – original draft, Writing – review & editing. JAM: Conceptualization, Data curation, Formal analysis, Funding acquisition, Investigation, Methodology, Project administration, Resources, Software, Supervision, Validation, Visualization, Writing – original draft, Writing – review & editing.
