# Supplementary material for: COVID-19 mortality rate and its determinants in Ethiopia: a systematic review and meta-analysis
Source: Front Med (Lausanne). 2024 Feb 27;11:1327746. doi: 10.3389/fmed.2024.1327746 (PMC10928001; doi:10.3389/fmed.2024.1327746)
Supplement: Supplementary file 2 [file Table_2.DOCX]

S2 File. Comprehensive search strategy for COVID-19 mortality in Ethiopia

| **Databases** | **Key search terms or phrases** |
| --- | --- |
|  | ((((((((((((((((((((((((((((((((((((((((((Mortality[MeSH Terms]) OR (Mortality)) OR ("Mortalities")) OR ("Mortality Rate")) OR ("Mortality Rates")) OR ("Rate Mortality")) OR ("Death Rate")) OR ("Death Rates")) OR ("Rate Death")) OR ("Case Fatality Rate")) OR ("Case Fatality Rates")) OR ("Rate Case Fatality")) OR ("Rates Case Fatality")) OR ("CFR Case Fatality Rate")) OR ("Crude Death Rate")) OR ("Crude Death Rates")) OR ("Death Rate Crude")) OR ("Rate Crude Death")) OR ("Crude Mortality Rate")) OR ("Crude Mortality Rates")) OR ("Mortality Rate Crude")) OR ("Rate Crude Mortality")) OR ("Decline Mortality")) OR ("Mortality Declines")) OR ("Mortality Decline")) OR ("Mortality Determinants")) OR ("Determinants Mortality")) OR ("Determinant Mortality")) OR ("Mortality Determinant")) OR ("Mortality Differential")) OR ("Differential Mortality")) OR ("Differential Mortalities")) OR ("Age-Specific Death Rate")) OR ("Age-Specific Death Rates")) OR ("Death Rate Age-Specific")) OR ("Rate Age-Specific Death")) OR ("Age Specific Death Rate")) OR ("Mortality Excess")) OR ("Excess Mortality")) OR ("Excess Mortalities") AND ((2020/1/1:2024/1/22[pdat]) AND (english[Filter]))) AND ((((((((((((((((((((((((((((((((((((((("COVID-19"[MeSH Terms]) OR ("COVID-19")) OR ("COVID 19")) OR ("2019-nCoV Infection")) OR ("2019 nCoV Infection")) OR ("2019-nCoV Infections")) OR ("Infection 2019-nCoV")) OR ("SARS-CoV-2 Infection")) OR ("Infection SARS-CoV-2")) OR ("SARS CoV 2 Infection")) OR ("SARS-CoV-2 Infections")) OR ("2019 Novel Coronavirus Disease")) OR ("2019 Novel Coronavirus Infection")) OR ("COVID-19 Virus Infection")) OR ("COVID 19 Virus Infection")) OR ("COVID-19 Virus Infections")) OR ("Infection COVID-19 Virus")) OR ("Virus Infection COVID-19")) OR ("COVID19")) OR ("Coronavirus Disease 2019")) OR ("Disease 2019 Coronavirus")) OR ("Coronavirus Disease-19")) OR ("Coronavirus Disease 19")) OR ("Severe Acute Respiratory Syndrome Coronavirus 2 Infection")) OR ("COVID-19 Virus Disease")) OR ("COVID 19 Virus Disease")) OR ("COVID-19 Virus Diseases")) OR ("Disease COVID-19 Virus")) OR ("Virus Disease COVID-19")) OR ("SARS Coronavirus 2 Infection")) OR ("2019-nCoV Disease")) OR ("2019 nCoV Disease")) OR ("2019-nCoV Diseases")) OR ("Disease 2019-nCoV")) OR ("COVID-19 Pandemic")) OR ("COVID 19 Pandemic")) OR ("COVID 19 Pandemic")) OR ("Pandemic COVID-19")) OR ("COVID-19 Pandemics") AND ((2020/1/1:2024/1/22[pdat]) AND (english[Filter])))) AND (((Adult[MeSH Terms]) OR (Adult)) OR (Adults) AND ((2020/1/1:2024/1/22[pdat]) AND (english[Filter])))) AND ((Ethiopia[MeSH Terms]) OR (Ethiopia) AND ((2020/1/1:2024/1/22[pdat]) AND (english[Filter]))) |
| **PubMed** |  |
| Total articles | 57 |
| **CINAHL** | Mortality Subject AND COVID-19 Subject AND Ethiopia Subject |
| Total articles | 37 |
| **Google scholar** | "COVID-19 Mortality rate" determinants Ethiopia |
| Total articles | 208 |
| **African Journals Online** | COVID-19 mortality rate and its determinants in Ethiopia |
| Total articles | 308 |
| **Total articles retrieved from all databases** | **610** |
