# Supplementary material for: COVID-19 mortality rate and its determinants in Ethiopia: a systematic review and meta-analysis
Source: Front Med (Lausanne). 2024 Feb 27;11:1327746. doi: 10.3389/fmed.2024.1327746 (PMC10928001; doi:10.3389/fmed.2024.1327746)
Supplement: Supplementary file 3 [file Table_3.DOCX]

S3 File. Data extraction format for COVID-19 mortality in Ethiopia

| ID | Authors, publication year | Region | Study design | Study period | Sample size (N) | Cases (n) | | IR | PD |
| --- | --- | --- | --- | --- | --- | --- | --- | --- | --- |
|  |  |  |  |  |  | n | % |  |  |
|  | Kaso AW, et al. (39), 2022 | Oromia | Retrospective | 2020-2021 | 308 | 50 | 16.2 | - | - |
|  | Kaso AW, et al.(40), 2022 | Oromia | Retrospective | 2020-2021 | 422 | 47 | 11.13 | 6.35 | 1000 |
|  | Birhanu A, et al.(55), 2022 | Harari | Retrospective | 2020-2021 | 355 | 96 | 27.04 | - | - |
|  | Dessie AM,et al.(45), 2022 | Amhara | Retrospective | 2020-2021 | 28533 | 2873 | 10.07 | 11.78 | 1000 |
|  | Mengist B, et al.(46),2022 | Amhara | Retrospective | 2020-2021 | 522 | 29 | 5.56 | 4.7 | 1000 |
|  | Tamiru DH,et al. (47), 2023 | Amhara | Retrospective | 2020-2021 | 452 | 37 | 8.2 | - | - |
|  | Getahun GK, et al (54), 2023 | Addis Ababa | Retrospective | 2021 | 393 | 32 | 8.1 | - | - |
|  | Habtewold EM,et al.(38),2022 | Oromia | Prospective | 2021 | 852 | 97 | 11.4 | 9.9 | 1000 |
|  | Gudina EK, et al.(41), 2021 | Oromia | Retrospective | 2020 | 4398 | 52 | 1.2 | - | - |
|  | Kebede F, et al.(58), 2022 | Benishangul Gumuz | Retrospective | 2020 | 288 | 50 | 17.4 | 1.8 | 100 |
|  | Ayana GM, et al.(56), 2021 | Harari | Retrospective | 2020-2021 | 531 | 101 | 19.02 | 16.2 | 1000 |
|  | Churiso G, et al.(48), 2022 | SNNP | Retrospective | 2020-2021 | 220 | 49 | 22.3 | - | - |
|  | Nega G, et al.(53), 2022 | Addis Ababa | Retrospective | 2020-2021 | 496 | 314 | 63.3 | 56.7 | 1000 |
|  | Abebe HT,et al.(57), 2022 | Tigray | Retrospective | 2020 | 139 | 56 | 40.3 | - | - |
|  | Lemma Tirore L, et al.(49),2022 | SNNP | Retrospective | 2020-2021 | 845 | 70 | 8.3 | - | - |
|  | Misganaw S, et al.(50),2023 | SNNP | Retrospective | 2020-2021 | 1032 | 128 | 12.4 | - | - |
|  | Tsegaye S,et al.(42), 2022 | Oromia | Retrospective | 2020-2022 | 300 | 13 | 4.3 | - | - |
|  | Tolossa T, et al.(43), 2021 | Oromia | Retrospective | 2020 | 263 | 15 | 5.7 | - | - |
|  | Tolossa T,et al.(44),2022 | Oromia | Retrospective | 2020-2021 | 318 | 51 | 16.04 | 14.1 | 1000 |
|  | Atamenta T, et al.(52),2023 | Addis Ababa | Retrospective | 2020 | 602 | 87 | 14.5 | 10.7 | 1000 |
|  | Fantaw S, et al. (38), 2023 | SNNP | Retrospective | 2020-2022 | 1038 | 181 | 17.4 | - | - |

SNNP = Southern Nation Nationality People. PD: person day, IR: incidence rate, Dash (-) indicates the data is not available or has not been reported
