# Supplementary material for: COVID-19 mortality rate and its determinants in Ethiopia: a systematic review and meta-analysis
Source: Front Med (Lausanne). 2024 Feb 27;11:1327746. doi: 10.3389/fmed.2024.1327746 (PMC10928001; doi:10.3389/fmed.2024.1327746)
Supplement: Supplementary file 4 [file Table_4.DOCX]

# S4 File. The quality of included studies as determined by the Newcastle-Ottawa Scale Appraisal Checklist.

| **For retrospective cohort study design** | | | | | | | | | | | | Quality of the study |
| --- | --- | --- | --- | --- | --- | --- | --- | --- | --- | --- | --- | --- |
| Author, Publication year | Selection | | | | | | Comparability | Outcome | | | Total score  (100%) |  |
|  | 1 | 2 | 3 | | | 4 |  | 1 | 2 | 3 |  |  |
| Kaso AW, et al. (39), 2022 | Y | Y | Y | | | Y | N | Y | NA | NA | 5/7 (71.4%) | high |
| Kaso AW, et al.(40), 2022 | Y | Y | Y | | | Y | Y | Y | NA | NA | 7/7/ (100%) | high |
| Birhanu A, et al.(55), 2022 | Y | Y | y | | | Y | N | Y | NA | NA | 5/7 (71.4%) | high |
| Dessie AM,et al.(45), 2022 | Y | Y | Y | | | Y | Y | Y | NA | NA | 7/7 (100%) | high |
| Mengist B, et al.(46),2022 | Y | Y | Y | | | Y | Y | Y | NA | NA | 7/7 (100%) | high |
| Tamiru DH,et al.(47), 2023 | Y | Y | Y | | | Y | N | Y | NA | NA | 5/7 (71.4%) | high |
| Getahun GK, et al (54),2023 | Y | Y | Y | | | Y | Y | Y | NA | NA | 7/7(100%) | high |
| Gudina EK, et al.(41), 2021 | Y | Y | Y | | | Y | N | Y | NA | NA | 5/7 (71.4%) | high |
| Kebede F, et al.(58), 2022 | Y | Y | Y | | | Y | Y | Y | NA | NA | 7/7 (100%) | high |
| Ayana GM, et al.(56), 2021 | Y | Y | Y | | | Y | Y | Y | NA | NA | 7/7 (100%) | high |
| Churiso G, et al.(48), 2022 | Y | Y | Y | | | Y | N | Y | NA | NA | 5/7 (71.4%) | high |
| Nega G, et al.(53), 2022 | Y | Y | Y | | | Y | Y | Y | NA | NA | 7/7 (100%) | high |
| Abebe HT,et al.(57), 2022 | Y | Y | Y | | | Y | N | Y | NA | NA | 5/7 (71.4%) | high |
| Lemma Tirore L, et al.(49),2022 | Y | Y | Y | | | Y | N | Y | NA | NA | 5/7 (71.4%) | high |
| Misganaw S, et al.(50),2023 | Y | Y | Y | | | Y | y | Y | NA | NA | 7/7 (100%) | high |
| Tsegaye S,et al.(42), 2022 | Y | Y | Y | | | Y | N | Y | NA | NA | 5/7 (71.4%) | high |
| Tolossa T, et al.(43), 2021 | Y | Y | Y | | | Y | N | Y | NA | NA | 5/7 (71.4%) | high |
| Tolossa T,et al.(44),2022 | Y | Y | Y | | | Y | Y | Y | NA | NA | 7/7 (100%) | high |
| Atamenta T, et al.(52),2023 | Y | Y | Y | | | Y | Y | Y | NA | NA | 7/7 (100%) | high |
| Fantaw S, et al.(51), 2023 | Y | Y | Y | | | Y | N | Y | NA | NA | 5/7(71.4%) | high |
| **For prospective cohort study design** | | | | | | | | | | | |  |
| Author, Publication year | Selection | | | | | | Comparability | Outcome | | | Total score  (100%) |  |
|  | 1 | 2 | | 3 | 4 | |  | 1 | 2 | 3 |  |  |
| Habtewold EM,et al.(39),2022 | Y | Y | | Y | Y | | Y | Y | Y | Y | 9/9 (100%) | high |

The quality score of <50%=low quality, 50%-69%=medium quality, ≥70%=high quality

**Descriptions**

| **Assessment of quality of a cohort study – Newcastle Ottawa Scale** |  |
| --- | --- |
| 1. **Selection (4 points),** (**NB exposure = intervention**) |  |
| 1. Representativeness of the intervention cohorta) Truly representative of the average, elderly, community-dwelling resident ★b) Somewhat representative of the average, elderly, community-dwelling resident ★c) Selected group of patients, e.g. only certain socio-economic groups/areasd) No description of the derivation of the cohort | 🞏  🞏  🞏  🞏 |
| 2. Selection of the non-intervention cohorta) Drawn from the same community as the intervention cohort ★b) Drawn from a different sourcec) No description of the derivation of the non-intervention cohort | 🞏  🞏  🞏 |
| 3. Ascertainment of interventiona) Secure record (e.g. health care record) ★b) Structured interview ★c) Written self-reportd) Other / no description | 🞏  🞏  🞏  🞏 |
| 4. Demonstration that outcome of interest was not present at start of studya) Yes ★b) No | 🞏  🞏 |
| Comparability (maximum 2 points) |  |
| 1. Comparability of cohorts on the basis of the design or analysisa) Study controls for age, sex, marital status ★b) Study controls for any additional factors (e.g. socio-economic status, education) ★ | 🞏  🞏 |
| Outcome (3 points) |  |
| 1. Assessment of outcomea) Independent blind assessment ★b) Record linkage ★c) Self-report d) Other / no description | 🞏  🞏  🞏  🞏 |
| 2. Was follow up long enough for outcomes to occura) Yes, if median duration of follow-up >= 6 month ★b) No, if median duration of follow-up < 6 months | 🞏  🞏 |
| 3. Adequacy of follow up of cohortsa) Complete follow up: all subjects accounted for ★b) Subjects lost to follow up unlikely to introduce bias: number lost <= 20%, ★ or description of those lost suggesting no different from those followedc) Follow up rate < 80% (select an adequate %) and no description of those lostd) No statement | 🞏  🞏  🞏  🞏 |
